# Withdrawn: Eco‐evolutionary factors that influence its demographic oscillations in *Prochilodus costatus* (Actinopterygii: Characiformes) populations evidenced through a genetic spatial–temporal evaluation

**DOI:** 10.1111/eva.12950

**Published:** 2023-02-27

Withdrawal: Sandra Ludwig, Juliana Martins Pimentel, Leonardo Cardoso Resende, Evanguedes Kalapothakis, Eco‐evolutionary factors that influence its demographic oscillations inProchilodus costatus (Actinopterygii: Characiformes) populations evidenced through a genetic spatial‐temporal evaluation, Evolutionary Applications 2020 (https://doi.org/10.1111/eva.12950).

The above article, published online on 11 March 2020 in Wiley Online Library as an Accepted Article, has been withdrawn by agreement between the authors, journal Editor‐in‐Chief Louis Bernatchez and John Wiley and Sons Ltd. The withdrawal has been agreed because the authors are unable to finalize the APC payment for their article for publication in the journal as the Version of Record.

